# Wearable, stable, highly sensitive hydrogel–graphene strain sensors

**DOI:** 10.3762/bjnano.10.47

**Published:** 2019-02-14

**Authors:** Jian Lv, Chuncai Kong, Chao Yang, Lu Yin, Itthipon Jeerapan, Fangzhao Pu, Xiaojing Zhang, Sen Yang, Zhimao Yang

**Affiliations:** 1 School of Science, MOE Key Laboratory for Non-Equilibrium Synthesis and Modulation of Condensed Matter, Xi’an Jiaotong University, Xi’an 710049, Shaanxi, P. R. China; 2 Department of NanoEngineering, University of California, San Diego, La Jolla, California 92093, USA; 3 Department of Chemistry, Faculty of Science, Prince of Songkla University, Hat Yai, Songkhla 90112, Thailand; 4 Collaborative Innovation Center of Suzhou Nano Science and Technology, Xi’an Jiaotong University Suzhou Academy, Suzhou 21500, P. R. China; 5 Research institute of Xi'an Jiaotong University, Hangzhou, Zhejiang, 311215, P. R. China

**Keywords:** graphene, high sensitivity, hydrogel, strain sensor, wearable sensor

## Abstract

A stable and highly sensitive graphene/hydrogel strain sensor is designed by introducing glycerol as a co-solvent in the formation of a hydrogel substrate and then casting a graphene solution onto the hydrogel in a simple, two-step method. This hydrogel-based strain sensor can effectively retain water in the polymer network due to the formation of strong hydrogen bonding between glycerol and water. The addition of glycerol not only enhances the stability of the hydrogel over a wider temperature range, but also increases the stretchability of the hydrogel from 800% to 2000%. The enhanced sensitivity can be attributed to the graphene film, whereby the graphene flakes redistribute to optimize the contact area under different strains. The careful design enables this sensor to be used in both stretching and bending modes. As a demonstration, the as-prepared strain sensor was applied to sense the movement of finger knuckles. Given the outstanding performance of this wearable sensor, together with the proposed scalable fabrication method, this stable and sensitive hydrogel strain sensor is considered to have great potential in the field of wearable sensors.

## Introduction

Wearable, flexible sensors to monitor human body pressure, temperature, strain, and chemicals hold great potential in the field of bioelectronics, artificial intelligence, and soft robotics [1–2]. Among these sensors, strain sensors can translate an external applied tensile force into electrical signal, hence attracting numerous research efforts for health monitoring, biomechanics studies and artificial skin for soft robotics [3–4]. The current, state of the art strategy to fabricate flexible strain sensors involves the integration of a conductive film on an elastomeric polymer and the embedding of conductive materials into the polymer matrix [5–6]. However, the lack of seamless conformation to curvatures in human skin has impeded further integration as a wearable sensing component [7].

Hydrogels, with mechanical properties like biological tissues and consisting of three-dimensional polymer networks that can retain a large amount of water, can serve as ideal vehicles for wearable devices [8–9]. Several hydrogel-based strain sensors demonstrating high flexibility, self-healing properties and skin-attachable wearability, have been fabricated in the reported literature [2,10–11]. However, their inability to retain water over a long period largely prevents the hydrogel-based strain sensors from being widely used in a realistic environment with extended usage. Lu et al. utilized a water and glycerol binary-solvent system to produce a hydrogel with good thermal tolerance, while maintaining all the properties over a wide temperature range (−20 to 60 °C) [12]. To date, the use of a moisture-retaining hydrogel to fabricate soft, flexible strain sensors is rarely reported. Another issue to be solved is related to increasing the sensitivity of the hydrogel strain sensor as the ionically conductive hydrogel exhibits low resistance changes with applied strain [13].

In this study, a flexible, stable, high-sensitivity, graphene-based, water/glycerol (WG) binary-solvent hydrogel (graphene/WG-hydrogel) strain sensor is designed via a two-step method. Water and glycerol are used as solvents to synthesize the hydrogel substrate with long-lasting moisture-retaining properties [12]. The continuous graphene film is cast onto the hydrogel through drop casting and drying. Although the bare hydrogel already shows resistance changes with respect to strain, the graphene film was used to further increase the sensitivity. The graphene/WG-hydrogel strain sensor can be used to sense human finger movements. This stable, soft, high-sensitivity hydrogel strain sensor shows great promise for the development of applicable, wearable strain sensors.

## Experimental

**Chemicals:** Acrylic acid (C_3_H_4_O_2_), ammonium persulfate ((NH_4_)_2_S_2_O_8_), α-ketoglutarate (C_5_H_6_O_5_), glycerol (C_3_H_8_O_3_), acrylamide (C_3_H_5_NO) and methylene-bis-acrylamide (C_7_H_10_N_2_O_2_) were purchased from Aladdin (Shanghai, China). The 2 mg/mL aqueous graphene dispersion was purchased from Tanfeng Graphene (Suzhou, China). Silver epoxy was purchased from Ted Pella (Redding, USA).

**Formulation of the hydrogel:** Acrylic acid, acrylamide, ammonium persulfate, methylene-bis-acrylamide and α-ketoglutarate were mixed in a beaker containing glycerol/water (1:3 v/v) binary solvent with concentrations of 40 mg/mL, 200 mg/mL, 25 mg/mL, 0.15 mg/mL, and 0.3 mg/mL, respectively, to make the monomer solution. Then the solution was mixed by magnetic stirring for 1 h. After the mixed solution was poured into the mold and irradiated by the UV light for 1 h, the hydrogel substrate was formed. The pure-water hydrogel was formulated in the same way as the binary solvent hydrogel, except that no glycerol was added.

**Formulation of the graphene/hydrogel:** The graphene film was cast on the hydrogel film using a drop casting and drying process. 5 mL of the 2 mg/mL graphene dispersion was drop casted onto the hydrogel and put in the oven at 35 °C until the graphene was fully dried.

**Assembly of the strain sensor:** The strain sensor was fabricated by connecting the graphene/hydrogel to a copper foil using the silver epoxy. The size of the graphene/hydrogel sensor is 5 × 30 mm.

**Characterization of the hydrogel and graphene/hydrogel:** The mechanical properties of the hydrogel were characterized in an electronic universal testing machine (CMT6503, Sans, Shenzhen, China). A field-emission scanning electron microscope (SEM, JSM-7000F, JEOL, Japan) was used for electron microimaging. The resistance changes of the strain sensor under stretching was monitored by a multimeter (Keithley 2400 Source Meter).

## Results and Discussion

**Figure 1:**
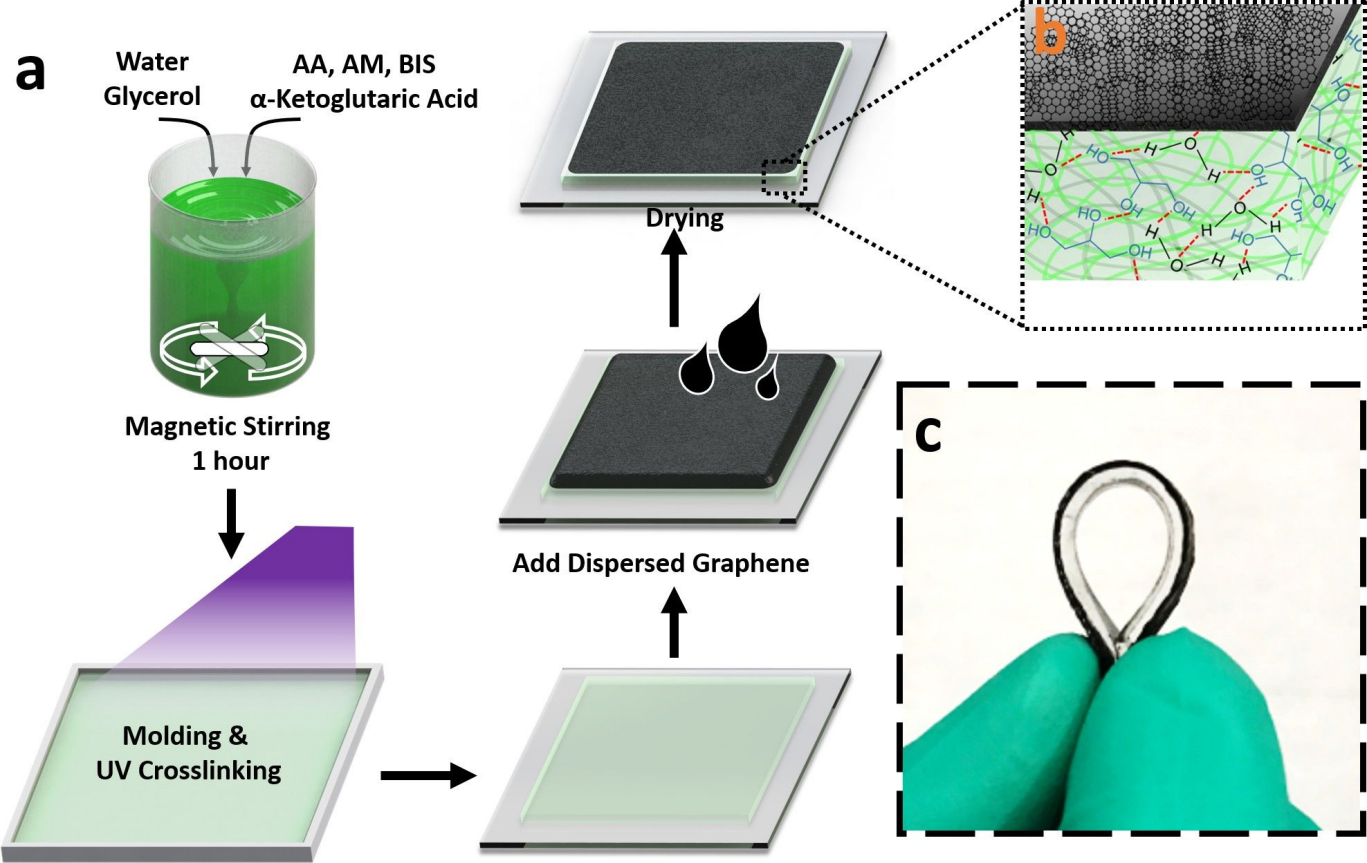
(a) Schematic of the preparation of the graphene/water-glycerol (WG) hydrogel composite material. (b) The double-layer composition of the graphene/WG-hydrogel composite material. (c) Photograph of the as-prepared flexible graphene/WG-hydrogel.

Figure 1a shows the two-step fabrication process of the graphene/WG-hydrogel composite material. The hydrogel, which is capable of withstanding relatively high temperature, was synthesized by the copolymerization of acrylic acid (AA) and acrylamide (AM) monomer in the water/glycerol solvent system under irradiation with UV light. A common problem of the reported hydrogels is the lack of long term stability, due to the tendency of the material to lose water at high temperature and freeze at low temperature [14]. Glycerol is a commonly used, nontoxic antifreeze additive. Here, hydrogen bonding between glycerol and water competes with hydrogen bonding between the water molecules, and the formation of ice at low temperature is restricted and the evaporation of water at high temperature is prohibited [11].

The graphene layer was formed on the hydrogel by directly drop casting the graphene solution onto the hydrogel and drying in an oven at 35 °C. The drying process removes the extra water in the graphene dispersion without removing the water inside the hydrogel, thus maintaining the mechanical properties of the material. The graphene layer serves to significantly increase the resistance change of the hydrogel under strain because of the spacing variations among graphene flakes. The schematic structure and a photograph of the composite electrode is shown in Figure 1b and Figure 1c, respectively.

**Figure 2:**
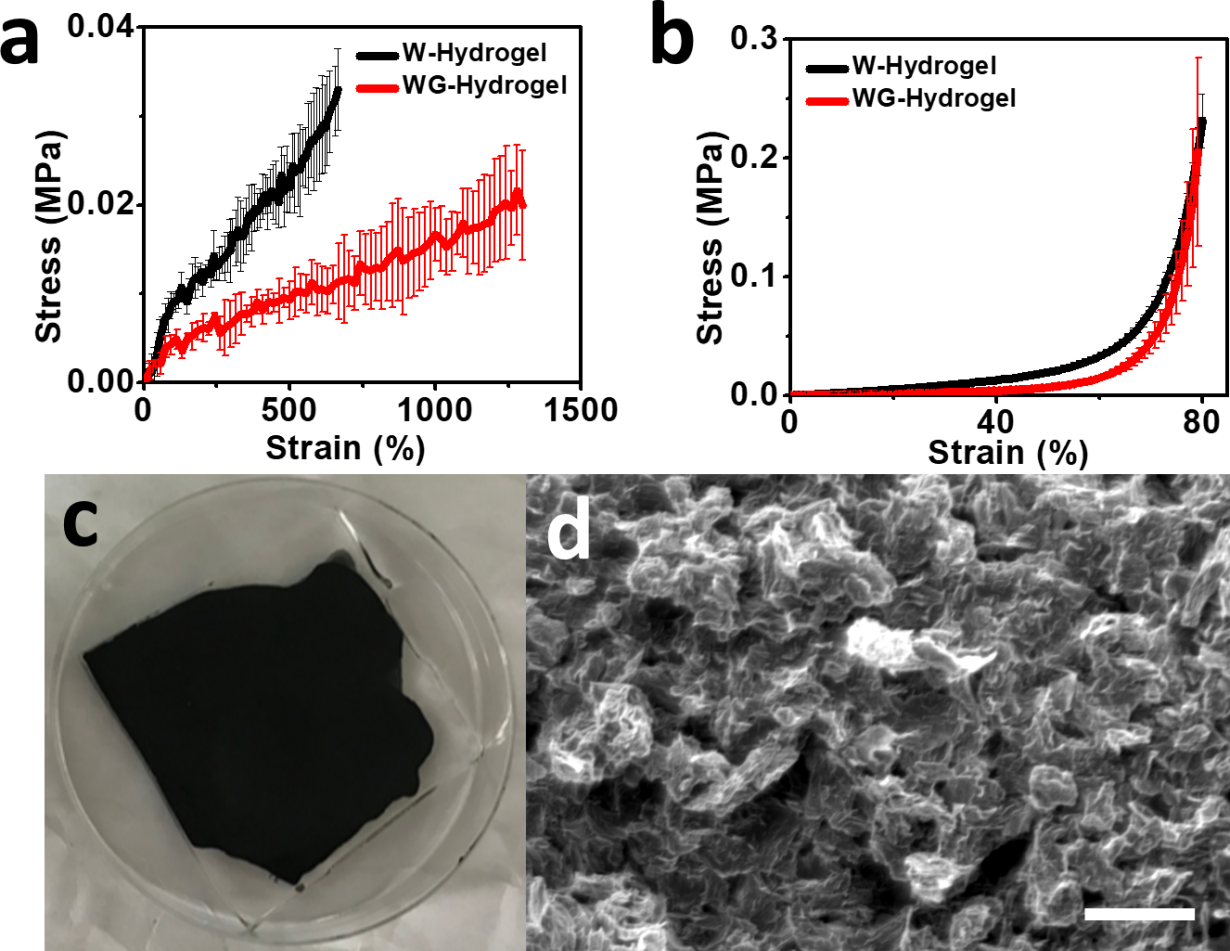
(a) The uniaxial tensile and (b) compressive strain–stress curves of the W-hydrogel and WG-hydrogel, respectively (*n* = 3). (c) The top view of the graphene/WG-hydrogel composite material. (d) An SEM image of the graphene film. Scale bar: 10 µm.

Besides serving to retain water in the hydrogel, the presence of glycerol also improves the mechanical performance of the hydrogel, as shown in Figure 2a and 2b. Figure 2a shows the uniaxial tensile strain–stress curves of the hydrogels fabricated with (WG-hydrogel) and without glycerol (W-hydrogel). Under the same strain, the WG-hydrogel shows less stress in the two strain–stress curves, indicating that including the glycerol provides the hydrogel with higher softness, thus making it more adaptable to external force. The complete yield strain–stress curves of the two hydrogels is shown in Supporting Information File 1, Figure S1, in which the fracture strain of the W-hydrogel is around 800% with a stress of 0.03 MPa, while the introduction of glycerol increases the break stress to 0.06 MPa at a strain of 2000%. The difference between the two hydrogels in the compressing strain–stress curve is not much as that in tensile mode, as shown in Figure 2b. An image of the synthesized graphene–hydrogel electrode is shown in Figure 2c. A uniform film is formed on the surface of the hydrogel after the drying process in the oven, which serves to increase the conductivity of the hydrogel. More details of the as-prepared graphene can be seen in the SEM image shown in Figure 2d. The graphene layer shows a randomly interconnected structure which allows the graphene flakes to slide when the layer is stretched, which will enhance the resistance change of the hydrogel under strain. The SEM image of the cross section of the graphene/hydrogel composite is shown in Supporting Information File 1, Figure S2. A great contact between the graphene layer and the hydrogel layer can be seen after the drying of the graphene solution.

**Figure 3:**
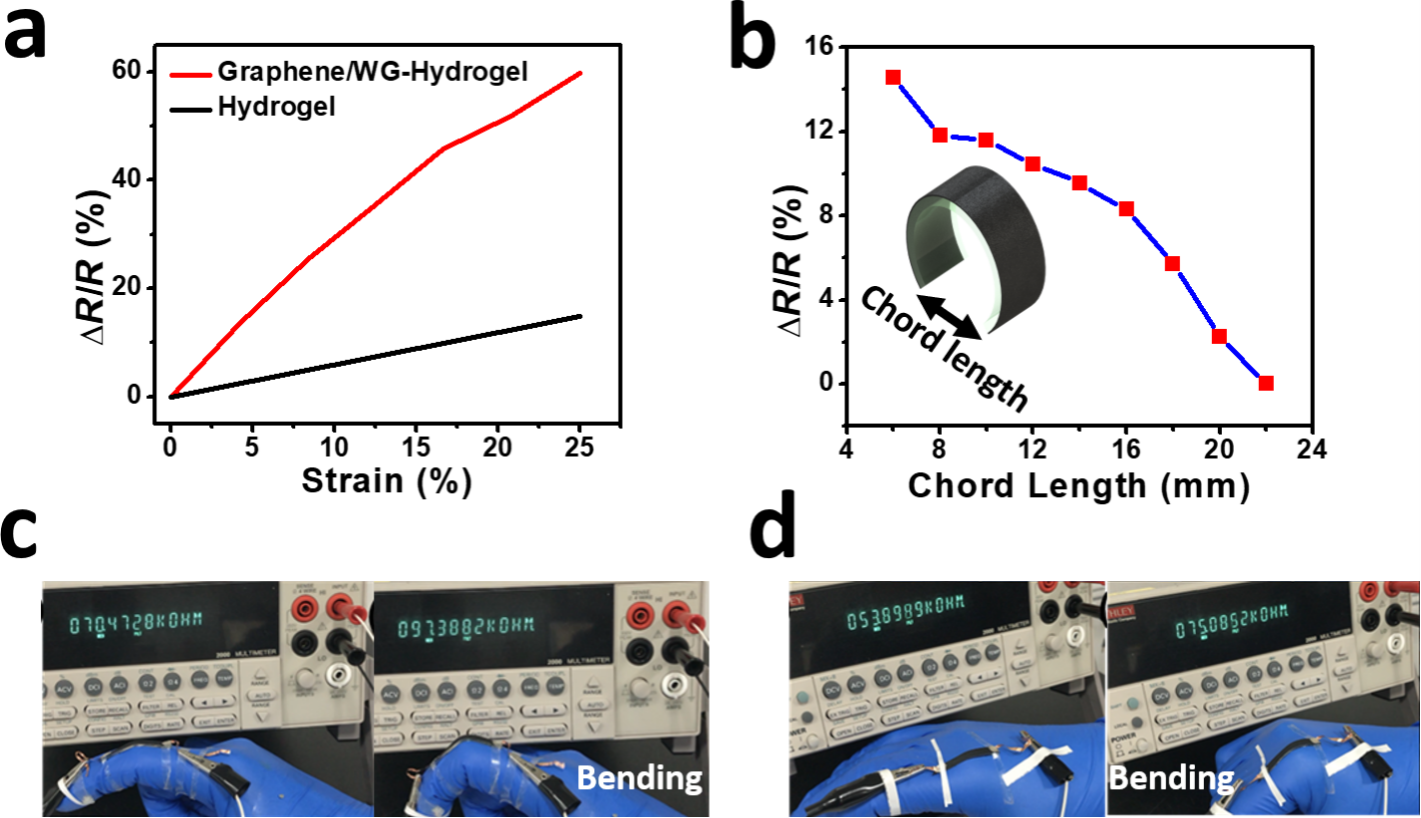
(a) The strain–resistance change curves of the WG-hydrogel and graphene/WG-hydrogel based strain sensors. (b) Resistance change of the graphene–hydrogel strain sensor versus chord length. Photographs showing the real life application of the strain sensor to sense the movement of the proximal interphalangeal joint (c) and the metacarpophalangeal joint (d).

The sensing performance of the graphene/WG-hydrogel composite material is shown in Figure 3a. The sensitivity of the graphene/WG-hydrogel composite sensor, represented by the gauge factor (the ratio of the relative electrical resistance change Δ*R*/*R* to the strain), is much higher than that of the WG-hydrogel sensor. At 25% stretching, the gauge factor of the graphene/WG-hydrogel is about 2.4, while that of the hydrogel is only about 0.59. This superior piezoresistive performance comes from two aspects: the intrinsic piezoresistive behavior of the WG-hydrogel and the electron conduction change of the graphene film under different contact conditions [15] (spacing variations and contact area under stretching). The ionic conductivity is responsible for the electrical conductivity of the bare WG-hydrogel. The hysteresis curve for graphene/WG-hydrogel strain sensor is illustrated in Supporting Information File 1, Figure S3. The stretching and releasing curve is almost symmetric, indicating that hysteresis is not obvious. Supporting Information File 1, Figure S4 shows optical images of the graphene/hydrogel composite before and after 10 times 25% stretching. It is clear that the bonding between the graphene layer and hydrogel layer is very firm, even after the stretching, suggesting the capability of the composite to endure cyclic stretching.

The graphene/WG-hydrogel strain sensor was also used to sense flexion, another type of human movement, as shown in Figure 3b. The outer curvature of the sensor experiences tensile strain and the inner curvature undergoes a compressive force when the graphene/hydrogel sensor is flexed, as shown in the inset of Figure 3b. The graphene film was set on the outer curvature, so that the graphene flakes are separated under the flexion. When the distance between two ends of the sensor was decreased from 22 mm to 6 mm, the resistance increased by 14.6%. To show the real workability of the graphene/WG-hydrogel strain sensor, the sensor was mounted on a nitrile glove worn on a human hand to sense the movement of finger knuckles. As shown in Figure 3c, two as-fabricated sensors were put on the proximal interphalangeal (PIP) joint and metacarpophalangeal (MCP) joint, respectively. Before, during and after bending, the graphene/WG-hydrogel sensors showed the expected changes, indicating the potential of our strain sensor to be used in a real working scenario.

## Conclusion

We have demonstrated a wearable, stable, and highly sensitive strain sensor, based on a binary solvent, graphene/WG-hydrogel composite material, synthesized using a two-step process. The long-term water-retention properties of the graphene/WG-hydrogel strain sensor can be attributed to the use of glycerol as a co-solvent. The hydrogel bonding between glycerol and water prevents water from being released from the polymer network, hence guaranteeing the long-term stability of the sensor. In addition, a graphene film is cast onto the WG-hydrogel to enhance the sensitivity of the hydrogel strain sensor. The strain sensor is demonstrated to operate in both stretching and flexuous modes, together with the ability to sense the movement of finger knuckles, suggesting the great potential of this soft and stable hydrogel-based strain sensor. In addition, the long-term moisture-retaining property of the WG-hydrogel provides an ideal substrate to cast other kinds of two-dimensional material films, such as MoS_2_, through a simple drop casting and drying method [16–18].

## Supporting Information

Yield strain stress curve of the hydrogels; Cross-section SEM image of the graphene/hydrogel composite; Hysteresis curve for the graphene/WG-hydrogel strain sensor; Optical cross-section images of the graphene/WG-hydrogel composite before and after stretching.

File 1 Additional figures.
