# Supplementary material for: Wearable, stable, highly sensitive hydrogel–graphene strain sensors
Source: Beilstein J Nanotechnol. 2019 Feb 14;10:475–80. doi: 10.3762/bjnano.10.47 (PMC6404424; doi:10.3762/bjnano.10.47)
Supplement: File 1 — Additional figures. [file Beilstein_J_Nanotechnol-10-475-s001.pdf]

BEILSTEIN JOURNAL OF NANOTECHNOLOGY

Supporting Information

for

Wearable, stable, highly sensitive hydrogel–graphene strain sensors

Jian Lv, Chuncai Kong, Chao Yang, Lu Yin, Itthipon Jeerapan, Fangzhao Pu, Xiaojing Zhang, Sen Yang and Zhimao Yang

Beilstein J. Nanotechnol. **2019,** *10,* 475–480. doi:10.3762/bjnano.10.47

Additional figures

License and Terms: This is a supporting information file under the terms of the Creative Commons Attribution License (http://creativecommons.org/licenses/by/4.0). Please note that the reuse, redistribution and reproduction in particular requires that the authors and source are credited.
The license is subject to the *Beilstein Journal of Nanotechnology* terms and conditions: (https://www.beilstein-journals.org/bjnano)

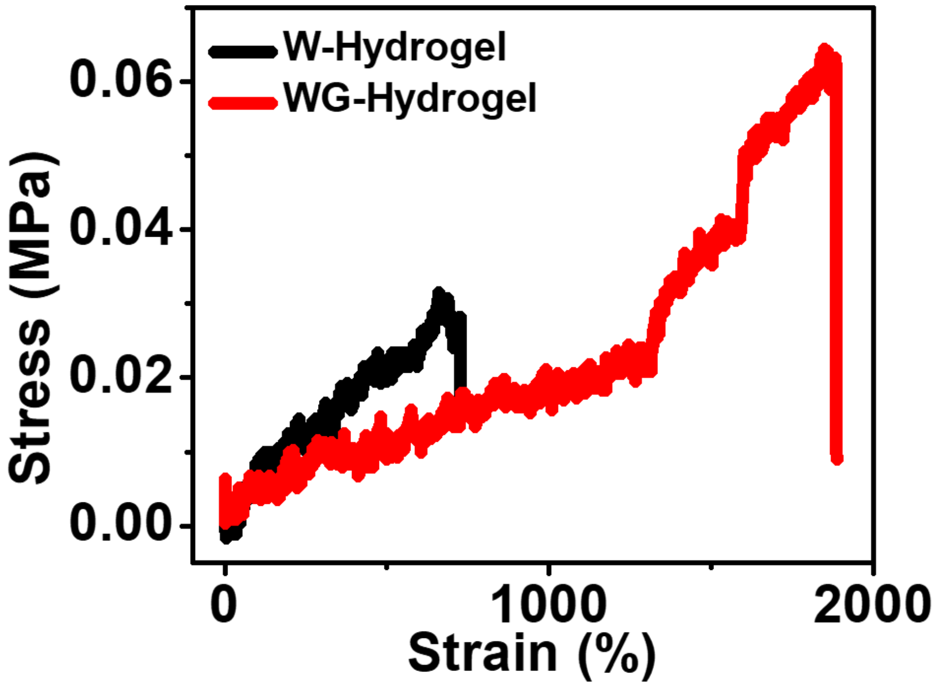

Figure S1: The yield strain-stress curves of the W-hydrogel and WG-hydrogel.

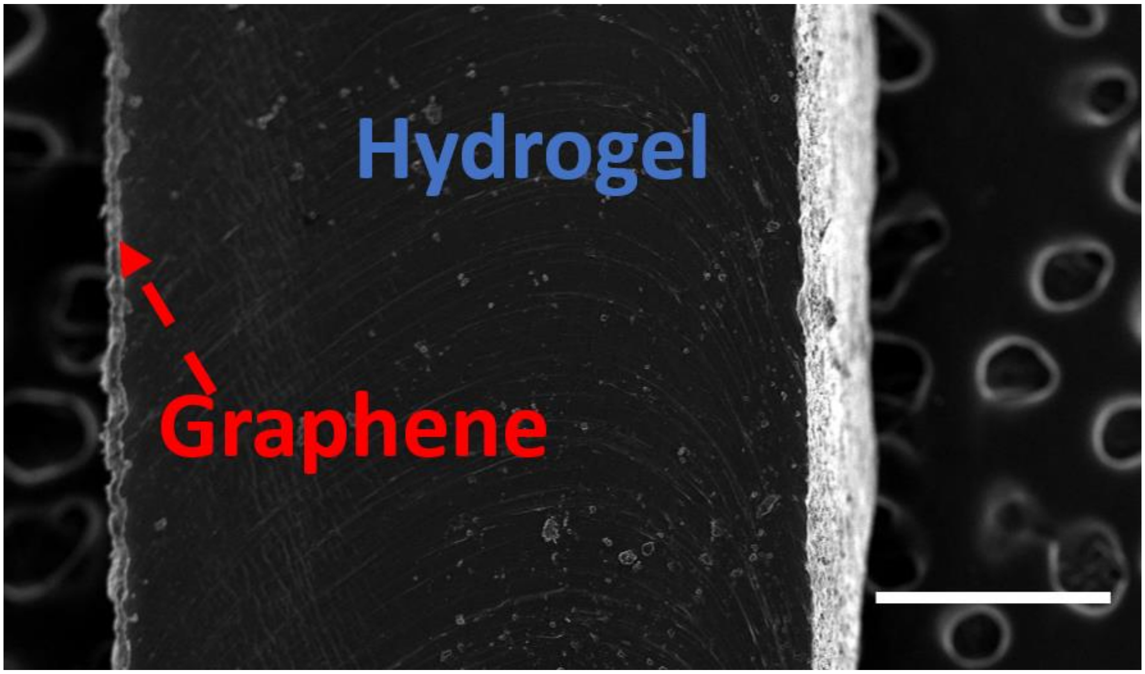

Figure S2: The cross-section SEM image of the graphene/hydrogel composite. Scale bar: 500 µm.

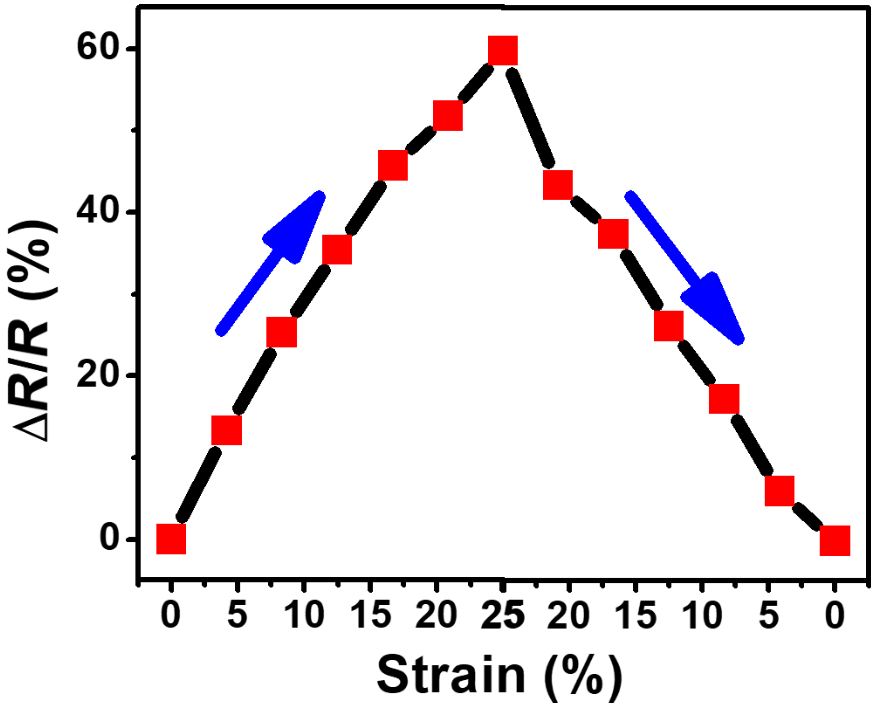

Figure S3: The hysteresis curve for graphene/WG-hydrogel strain sensor.

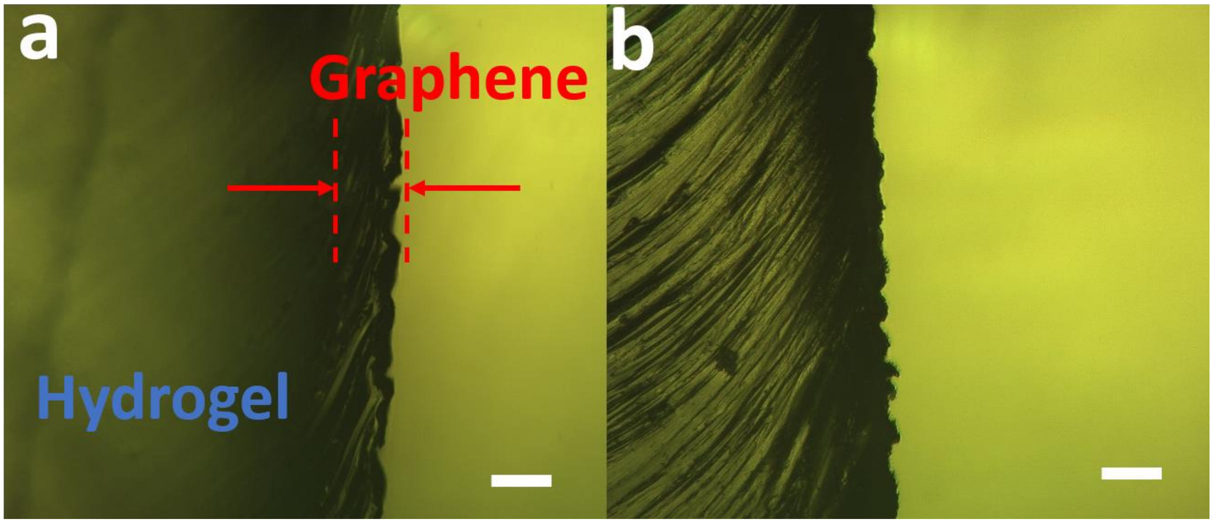

Figure S4: The optical cross-section images of the graphene/WG-hydrogel composite before (a) and after (b) 10 times of 25% stretching. Scar bar: 50 µm.
